# Case Report: A Case of Pituitary Adenoma Producing Growth Hormone and Thyroid-Stimulating Hormone Simultaneously

**DOI:** 10.3389/fendo.2021.659076

**Published:** 2021-03-22

**Authors:** Junpei Sanada, Fuminori Tatsumi, Shinji Kamei, Yoshiro Fushimi, Masashi Shimoda, Kenji Kohara, Shuhei Nakanishi, Kohei Kaku, Tomoatsu Mune, Hideaki Kaneto

**Affiliations:** Department of Diabetes, Endocrinology and Metabolism, Kawasaki Medical School, Kurashiki, Japan

**Keywords:** pituitary adenoma, growth hormone, thyroid-stimulating hormone, insulin-like hormone, immunostaining

## Abstract

**Background:**

Pituitary adenoma producing growth hormone (GH) or thyroid-stimulating hormone (TSH) is characterized by various specific symptoms and/or findings. However, the frequency of pituitary adenoma producing both hormones is relatively low. In this report, we show a case of pituitary adenoma producing both GH and TSH simultaneously.

**Case presentation:**

A 27-year-old woman was diagnosed as acromegaly based on various symptoms and clinical findings. For further examination and treatment, she was hospitalized in our institution. It was likely that this subject had pituitary adenoma producing both GH and TSH. In brain magnetic resonance imaging, there was a giant tumor around pituitary fossa. After the diagnosis of GH- and TSH-producing pituitary adenoma, pituitary tumor resection and cyber knife therapy were performed. In addition, we started additional treatment with somatostatin analog and GH receptor antagonist. After then, GH and insulin-like growth factor (IGF-1) levels were suppressed. After the operation, since thyroid function was not sufficiently suppressed, we started anti-thyroid drug thiamazole. After then, thyroid function was normalized and we stopped thiamazole. In GH and TSH staining, many GH-positive and TSH-positive cells were observed. These findings further confirmed our diagnosis that the pituitary adenoma in this subject produced both GH and TSH simultaneously.

**Conclusions:**

We should bear in mind the possibility of pituitary adenoma producing both GH and TSH at the same time.

## Introduction

Pituitary adenoma producing growth hormone (GHoma) is characterized by increased volume of limbs, lower jaw protrusion, macroglossia, increase of GH and IGF-1 levels, and the presence of pituitary adenoma in some image inspection. Pituitary adenoma producing thyroid-stimulating hormone (TSHoma) is characterized by various symptoms of hyperthyroidism such as palpitation, excessive sweating, hand tremor and decrease of body weight. In clinical practice, it is difficult to diagnose TSHoma at an early stage and it is often found after the appearance of visual field impairment. It is known that the frequency of pituitary adenoma producing both GH and TSH is relatively low (1, 2). In this report, we show a case of pituitary adenoma producing both GH and TSH at the same time.

## Case Report

A 27-year-old woman had persistent headache and amenorrhea. In addition, the size of her foot was increased by 0.5 cm for 6 months and lower jaw protrusion was observed. Thereby, she was referred to our institution. The data at that time were as follows: growth hormone (GH), 82.44 ng/ml; insulin-like growth factor-1 (IGF-1), 1,390 ng/ml; prolactin (PRL), 28.2 ng/ml. In addition, enlargement of pituitary fossa was observed in head X-ray, and thickening of heel pad was observed in foot X-ray. Based on these findings, she was diagnosed as acromegaly and hospitalized in our institution for further examination and treatment.

**Figure 1:**
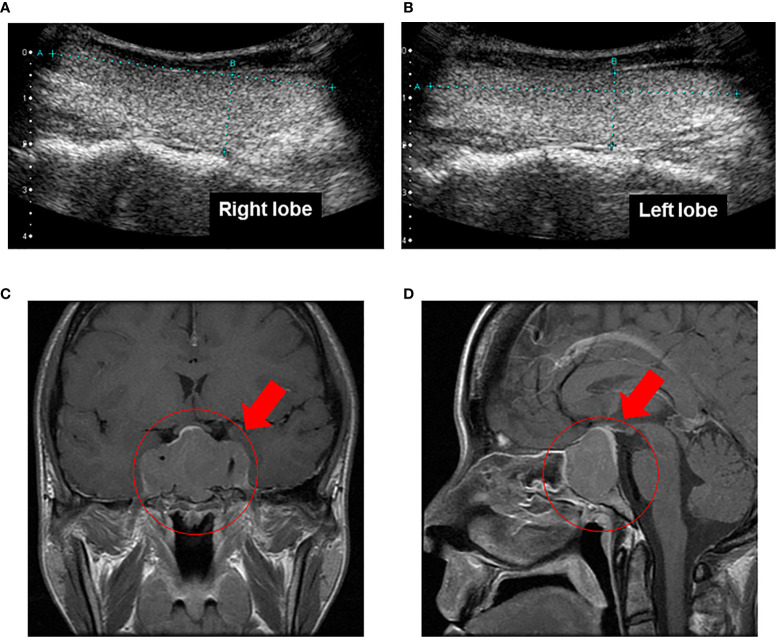
**(A, B)** In thyroid echography, both lobes of the thyroid gland were swelling although increase of blood flow was not observed. **(C, D)**. In brain magnetic resonance imaging, there was a giant tumor (51 × 34 × 22 mm) around pituitary fossa, pressuring on optic chiasm from the middle. Bilateral internal carotid arteries were surrounded by the tumor, and infiltration into the cavernous sinus was observed.

On admission, her height and body weight were 163.0 cm and 48.0 kg. Blood pressure and heart rate were 110/60 mmHg and 68 bpm. Body temperature was 37°C. Swelling of superciliary arch, hypertrophy of nose and lip, macroglossia, lower jaw protrusion, thickening of the plantar, enlargement of the palm and bitemporal hemianopsia were observed all of which were compatible with acromegaly. There were no abnormalities in heart and lung sound and in abdomen. The data on admission were as follows: GH, 51.12 ng/ml; IGF-1, 1,538.9 ng/ml; free triiodothyronine (FT3), 5.56 pg/ml (reference range: 2.3–4.3 pg/ml); free thyroxine (FT4), 2.52 ng/dl (0.9–1.7 ng/ml); TSH, 2.26 μU/ml. Other endocrine hormone levels were within normal range: adrenocorticotropic hormone (ACTH), 44.0 pg/ml; cortisol 8.5 μg/dl; dehydroepiandrosterone sulfate (DHEA-S), 375 μg/dl; plasma renin activity (PRA), 1.1 ng/ml/h; plasma aldosterone concentration (PAC), 29.7 pg/ml; luteinizing hormone (LH), 1.2 mU/ml; follicle stimulating hormone (FSH), 3.3 mU/ml; PRL, 26.2 ng/ml; estradiol, 26 pg/ml; testosterone 1.02 ng/ml. Thyroid-related antibodies including anti-Tg antibody, anti-TPO antibody and thyroid receptor antibody were all negative. Liver and renal function was within normal range. Inflammatory and tumor markers such as C-reactive protein (CRP), carcinoembryonic antigen (CEA) and colorectal carcinoma 19-9 (CA19-9) were also within normal range. In thyroid echography, both lobes of the thyroid gland were swelling although increase of blood flow was not observed (**Figures 1A, B** **)**. In brain magnetic resonance imaging (MRI), there was a giant tumor (51 × 34 × 22 mm) around pituitary fossa, pressuring on optic chiasm from the middle (**Figures 1C, D** **)**. Bilateral internal carotid arteries were surrounded by the tumor, and infiltration into the cavernous sinus was observed. Based on these findings, we finally diagnosed her as pituitary adenoma producing GH and TSH at the same time. After such diagnosis of GHoma and TSHoma, pituitary tumor resection was performed. Most of the tumor was resected during the pituitary surgery, although we fail to show what percentage of the tumor was resected.

**Figure 2:**
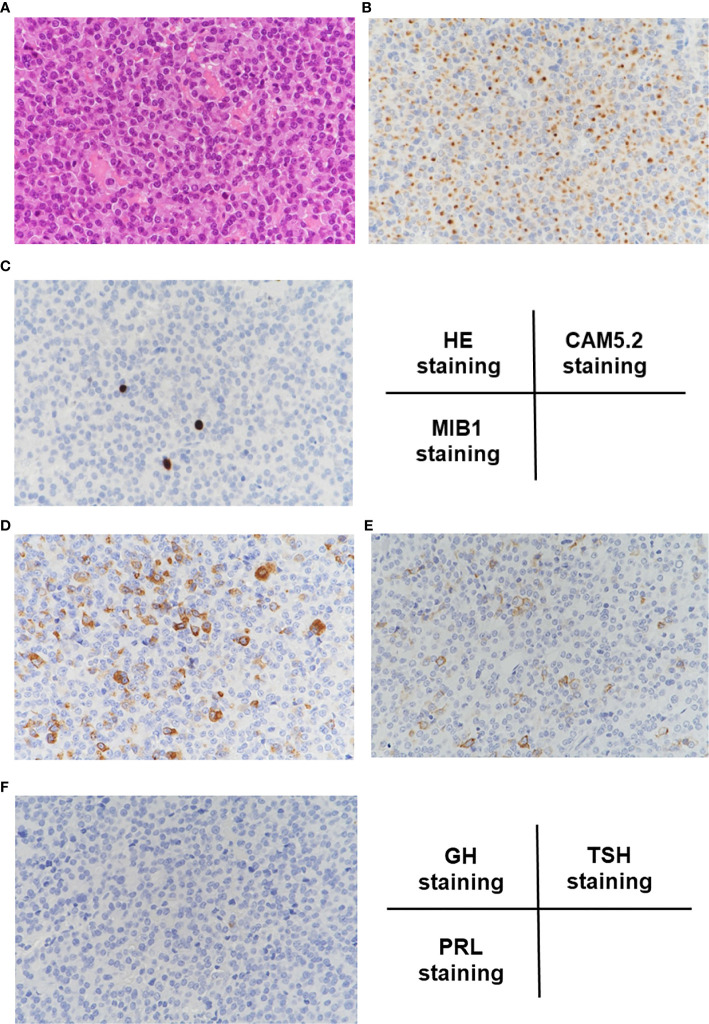
**(A)** In hematoxylin and eosin (HE) staining, solid proliferation of chromophobe cells was observed. **(B)** In CAM5.2 staining, many cytokeratin-producing cells were observed. **(C)** In MIB1 staining, percentage of MIB1-positive cells was about 3%, indicating that the proliferative activity was relatively low. **(D**–**F)** In GH staining, many GH-positive cells were observed, and in TSH staining, many TSH-positive cells were observed, whereas in prolactin staining, prolactin-positive cells were not detected at all.

In hematoxylin and eosin (HE) staining of the resected pituitary adenoma, solid proliferation of chromophobe cells was observed (**Figure 2A**). In CAM5.2 staining, many cytokeratin-producing cells were observed (**Figure 2B**). In MIB1 staining, percentage of MIB1-positive cells was about 3%, indicating that the proliferative activity was relatively low (**Figure 2C**). Based on these findings, she was pathologically diagnosed as sparsely granulated somatotroph adenoma. In GH staining, many GH-producing cells were observed (**Figure 2D**) and in TSH staining, many TSH-producing cells were observed (**Figure 2E**), whereas in prolactin staining, prolactin-producing cells were not detected at all (**Figure 2F**). These findings further confirmed our diagnosis that this subject had pituitary adenoma producing GH and TSH simultaneously.

**Figure 3:**
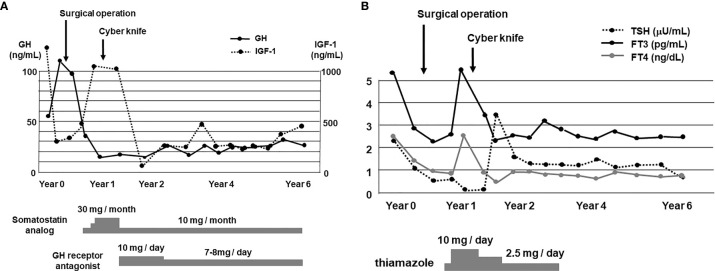
**(A)** Time course of GH and IGF-1 levels for 6 years. After the diagnosis of GHoma and TSHoma, pituitary tumor resection was performed. After about 1 year later, cyber knife therapy was performed together with the treatment with somatostatin analog and GH receptor antagonist. After these therapies, GH and IGF-1 levels were suppressed for a long period of time. **(B)** Time course of TSH, FT3 and FT4 levels for 6 years. After the operation, since thyroid function was not sufficiently suppressed, we started the treatment with anti-thyroid drug thiamazole. Since thyroid function was normalized after about 3 years later, we stopped the treatment with thiamazole. After then TSH, FT3 and FT4 levels were not increased for a long period of time.

**Figure 4:**
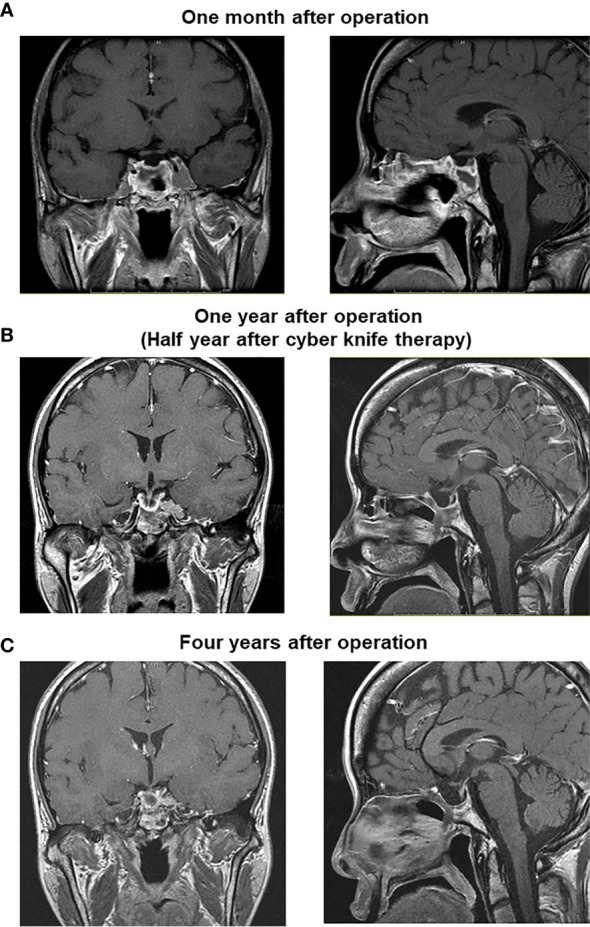
There were no particular abnormalities in brain MRI taken 1 month after operation **(A)**, 1 year after operation (half year after cyber knife therapy) **(B)** and 4 years after operation **(C)**.

After the surgery GH level was markedly decreased although it was still higher compared to its normal range (reference range in our institution: ≤2.47 ng/ml). After the surgery, IGF-1 level was also markedly decreased and became within normal range (reference range in our institution: 141–328 ng/ml) (**Figure 3A**). About 1 year later, IGF-1 level was increased again. After then, cyber knife therapy and treatment with somatostatin analog octreotide were performed. After the cyber knife therapy together with octreotide, IGF-1 was marked decreased and became within normal range (**Figure 3A**). However, since digestive symptoms were observed after the octreotide treatment, we started a combination therapy with GH receptor antagonist and octreotide based on the previous reports (3, 4). After then, GH and IGF-1 levels did not increase at least for 6 years (**Figure 3A**). After the operation, since thyroid function was not sufficiently suppressed, we started anti-thyroid drug thiamazole. After then, thyroid function was normalized and we stopped thiamazole. But TSH, FT3 and FT4 levels did not increase at least for 6 years (**Figure 3B**). In addition, there were no particular abnormalities in brain MRI taken 1 month after operation (**Figure 4A**), 1 year after operation (half year after cyber knife therapy) (**Figure 4B**) and 4 years after operation (**Figure 4C**).

## Discussion

In this report, we showed a case of pituitary adenoma producing both GH and TSH simultaneously. There are several reports showing plurihormonal pituitary adenoma (5–9). To the best of our knowledge, however, the frequency of pituitary adenoma producing both GH and TSH is relatively low. Indeed, it was reported that 45% of GH-producing pituitary adenoma produced GH alone but that 55% produced two or three kind of pituitary hormones (42% produced GH and prolactin, 7% produced GH and TSH, and 6% produced GH, TSH and prolactin) (10). In this sense, we think this case report is important and informative for many clinicians from the clinical point of view.

There are various reports about pituitary adenoma producing GH and TSH. For example, it was reported that GH- and TSH-producing pituitary adenoma had a larger maximum tumor diameter compared with pituitary adenoma secreting TSH alone. And the prognosis of patient with GH- and TSH-producing pituitary adenoma had a lower surgical complete remission rate and a worse prognosis (9, 10). These data indicate that postoperative drug administration would be necessary in many subjects with GH- and TSH-producing pituitary adenoma. Indeed, in our present case, post-operative drug administration such as somatostatin analog, GH receptor antagonist, and anti-thyroid drug was effective on the reduction and/or maintenance of GH, IGF-1 and TSH levels. In addition, it was reported about a case with GH- and TSH-producing pituitary adenoma together with papillary thyroid carcinoma. This case was successfully treated with octreotide and levothyroxine after thyroidectomy (6). Furthermore, it was reported about an extremely rare case with pituitary adenoma producing GH, TSH, prolactin and follicle-stimulating hormone (FSH). These four kinds of pituitary hormones were detected in immunostaining of the pituitary gland (7).

It is known that both GH-producing somatotroph and TSH-producing thyrotroph are derived from Pit-1-producing cells in the process of differentiation into mature pituitary cells (11). Therefore, from the point of cell lineage, GH-producing and TSH-producing cells are relatively close. Although speculative, we assume that such similarity in the differentiation process is associated with the formation of pituitary adenoma producing GH and TSH at the same time.

As shown in **Figure 3A**, since GH level was markedly decreased after the surgical operation, we think that this operation was effective on GHoma in this subject. Also, since IGF-1 level was markedly decreased after the cyber knife therapy and treatment with somatostatin analog, we think that these therapies were also effective on GHoma. However, since the cyber knife therapy and treatment with somatostatin analog were performed at the same time, it was difficult to separately assess the effect of cyber knife therapy and the effect of somatostatin analog. After these therapies, both GH and IGF-1 levels were not increased for a long period of time. These data indicate that these therapies exerted beneficial effects on GHoma in this subject.

As shown in **Figure 3B**, since TSH, FT3 and FT4 levels were decreased after the surgical operation, we think that this operation was effective on TSHoma in this subject. Also, since TSH and FT4 levels were markedly decreased after the cyber knife therapy and treatment of thiamazole, we think that these therapies were also effective on TSHoma. However, since the cyber knife therapy and treatment with thiamazole were performed at the same time, it was difficult to separately assess the effect of cyber knife therapy and the effect of thiamazole. After these therapies, TSH, FT3 and FT4 levels were not increased for a long period of time. These data indicate that these therapies exerted beneficial effects on TSHoma in this subject.

Although the tumor in this subject was giant, elevation of prolactin level was slight. Since we did not measure prolactin level in dilution, we failed to exclude the possibility of hook effect. In addition, if the pituitary tumor in this subject secreted prolactin, we think that dopamine receptor activator such as cabergoline might have been the mainstay of therapy in this subject.

There is a limitation in this case report. First, we failed to perform the magnified adjacent sections or mirror images of the pituitary adenoma which would have been very useful to show whether the same or different cells produce GH and TSH. As described above, both GH-producing somatotroph and TSH-producing thyrotroph are derived from Pit-1-producing cells in the process of differentiation. Therefore, although speculative, we assume that it is possible that the same pituitary cells secrete both hormones. Second, we failed to perform somatostatin receptors 2 and 5 immunostaining which would have been very useful in order to evaluate the responses to octreotide. Third, we failed to perform oral glucose tolerance test after the surgery.

Taken together, we experienced a case of pituitary adenoma producing both GH and TSH simultaneously. We should bear in mind the possibility of pituitary tumor producing both hormones at the same time especially in subjects with acromegaly.

## Data Availability Statement

The original contributions presented in the study are included in the article/supplementary material. Further inquiries can be directed to the corresponding author.

## Ethics Statement

Written informed consent was obtained from the individual for the publication of any potentially identifiable images or data included in this article.

## Author Contributions

JS, FT, SK, TM, and HK researched data and/or wrote the manuscript. YF, MS, KKo, SN, and KKa contributed to discussion. All authors contributed to the article and approved the submitted version.

## Conflict of Interest

The authors declare that the research was conducted in the absence of any commercial or financial relationships that could be construed as a potential conflict of interest.
